# A unique aspartyl protease gene expansion in *Talaromyces marneffei* plays a role in growth inside host phagocytes

**DOI:** 10.1080/21505594.2019.1593776

**Published:** 2019-04-11

**Authors:** Michael Payne, Harshini Weerasinghe, Irma Tedja, Alex Andrianopoulos

**Affiliations:** a Genetics, Genomics and Systems Biology, School of BioSciences, University of Melbourne, Australia; b School of Biotechnology and Biomolecular Sciences, University of New South Wales, Sydney, Australia

**Keywords:** *Talaromyces marneffei*, *Penicillium marneffei*, aspartyl protease, evolution, pathogenic fungi

## Abstract

Aspartyl proteases are a widely represented class of proteolytic enzymes found in eukaryotes and retroviruses. They have been associated with pathogenicity in a range of disease-causing microorganisms. The dimorphic human-pathogenic fungus *Talaromyces marneffei* has a large expansion of these proteases identified through genomic analyses. Here we characterize the expansion of these genes (*pop* – **p**aralogue **o**f ***p****ep*) and their role in *T. marneffei* using computational and molecular approaches. Many of the genes in this monophyletic family show copy number variation and positive selection despite the preservation of functional regions and possible redundancy. We show that the expression profile of these genes differs and some are expressed during intracellular growth in the host. Several of these proteins have distinctive localization as well as both additive and epistatic effects on the formation of yeast cells during macrophage infections. The data suggest that this is a recently evolved aspartyl protease gene family which affects intracellular growth and contributes to the pathogenicity of *T. marneffei*.

## Introduction

Proteases are a diverse and ubiquitously distributed group of enzymes that are important for a variety of biological processes including morphogenesis, cellular function, immunity, and nutrition. The aspartyl protease (aspartic peptidase) class of proteases similarly serve many roles but in particular have been implicated in virulence and pathogenicity of microorganisms such as HIV, *Plasmodium*, and *Candida* [1–3]. In several emerging and established fungal pathogens, genome sequencing has revealed unique expansions in the number of aspartyl protease-encoding genes and it is thought that this is commensurate with the shift towards a pathogenic lifestyle.

The acquisition or expansion of genes is a potential source of variation that can lead to the generation of new virulence factors. For example in the chytrid fungus *Batrachochytrium dendrobatidis*, responsible for amphibian infection and death worldwide, one aspartyl protease family is expanded to 77 genes (4.5 fold) relative to a closely related, less pathogenic, species [4]. Similarly, in the entomopathogenic genus *Metarhizium, M. anisopliae*, which has a broad host range, has significantly more aspartyl protease-encoding genes than its locust-specific relative, *M. acridum* [5]. In both instances the hypothesis is that this expansion plays an important role in virulence and pathogenicity; however, in neither case has this been tested functionally.

The most thoroughly studied families of aspartyl proteases belong to species in the *Candida* genus. These genes, known as secreted aspartyl proteases (SAPs), have been identified and/or characterized in *C. glabrata, C. tropicalis, C. dubliniensis* and *C. albicans* [3]. SAP genes were originally described in *C. albicans*, and several of the 10 members of this expansion have been associated with distinct cellular traits, including hyphal formation, adhesion, dimorphic switching and host cell apoptosis [6–8]. Despite some controversy due to complications with the genetic background of these mutants, and a revision of their importance to virulence in a murine model of hematogenously disseminated candidiasis [9], a number of these SAP genes have *bona fide* roles in immune responses, biofilm metabolism and mucosal infection [10–12]. In *C. parapsilosis*, production of three SAP proteins varies with respect to the tissue infected as well as between clinical isolates; thus multiple factors influence a single process such as infection [13,14].

*Aspergillus fumigatus* has two aspartyl protease-encoding genes and one of these, *pep1*, is expressed during infection in several animal models [15,16]. Mutant studies show that Pep1 is capable of degrading extracellular matrix proteins in the host, but loss does not affect tissue invasion or host mortality [17]. Therefore, while aspartyl proteases appear to have clear roles relevant to infection, both in species where these genes are expanded in number and those with only one to a few genes, loss does not always have a major impact on virulence. For both *Candida* species and *A. fumigatus*, this may be due to functional redundancy between members of the expanded families or other protease classes. An important distinction between *Candida* and many other morphologically complex fungi is that the latter also have other secreted protease classes besides the SAPs.

Despite these studies in *Candida* species and *A. fumigatus*, the function of aspartyl proteases in most other pathogens, including *T. marneffei* has not been examined. *T. marneffei* (formerly *Penicillium marneffei)* is a thermally dimorphic pathogen of humans that is endemic to Southeast Asia. The incidence of talaromycosis (formerly penicilliosis) peaked with the AIDS epidemic in this region [18]. *T. marneffei* is capable of growing as both multinucleate septate hyphae and uninucleate yeast cells dividing by fission [19]. The transition from hyphae to yeast and germination of conidia at 37°C *in vitro* occurs via an arthroconidial hyphal phase consisting of uninucleate, branched and septate cells. *In vivo* the transition from conidia to yeast cells is direct. The yeast form predominates during infection, where *T. marneffei* grows and proliferates within host phagocytic cells thereby avoiding exposure to, and clearance by, the immune system.

In this study, we describe a major expansion of aspartyl protease-encoding genes in *T. marneffei*, orthologous to the *A. fumigatus pep1* gene. The expansion, named **p**aralogue **o**f ***p****ep* (*pop*), was found to be monophyletic and is associated with repetitive elements and subtelomeric regions of the *T. marneffei* genome. This family shows evidence of significant positive selection as well as copy number variation amongst *T. marneffei* isolates. Despite these rapid evolutionary changes, the functional domains are conserved in the majority of the proteins. Based on their expression profiles, five *pop* genes were selected for functional analysis. Single and double deletions of these genes revealed cumulative and interdependent impacts on yeast cell formation during macrophage infection, while localization studies revealed unique cellular targeting during *in vitro* and *ex vivo* growth. The data suggest that the expansion of these genes may have been an important step in the evolution of pathogenicity in *T. marneffei*.

## Results

### The aspartyl protease expansion is monophyletic and variable

**Figure 1.**
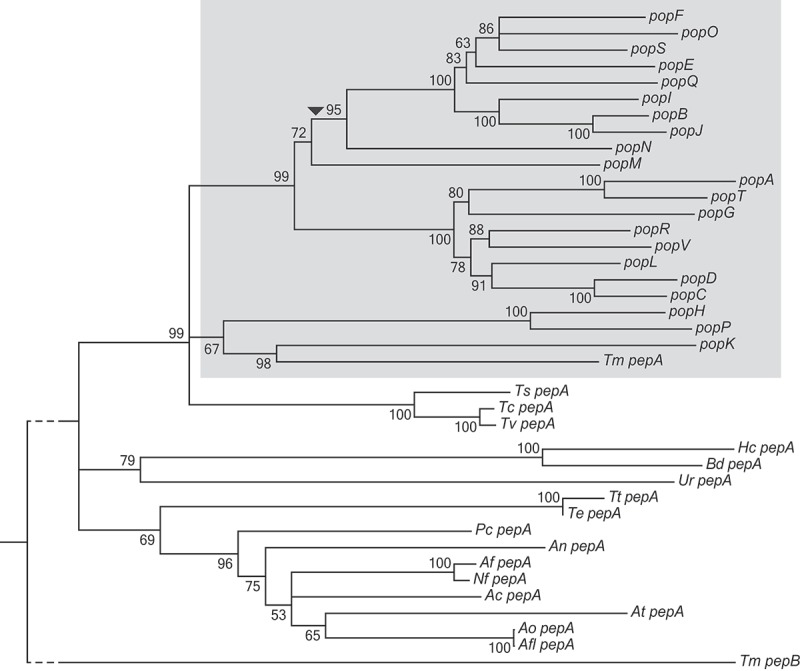
*T. marneffei* aspartyl protease encoding gene expansion. A neighbor-joining relatedness tree of genes in the *pop* expansion as well as *pepA* orthologues in other Eurotiomycetidae species. Branch confidence values are bootstrap supports from 1000 pseudoreplicates. *T. marneffei pepB* is included as an outgroup. The grey shading denotes expanded *pop* clade in *T. marneffei*. The dashed line denotes shortened branch lengths. The arrowhead indicates the position of a predicted retrotransposition event. Species abbreviations are as follows: *Ts, Talaromyces stipitatus; Tc, Talaromyces cellulolyticus; Tv, Talaromyces verruculosus; Te, Trichophyton equinum; Tt, Trichophyton tonsurans; An, Aspergillus nidulans; Pc, Penicillium chrysogenum; Af, Aspergillus fumigatus; Nf, Neosartorya fischeri; Ac, Aspergillus clavatus; At, Aspergillus terreus; Ao, Aspergillus oryzae; Aa, Aspergillus avus; Ur, Uncinocarpus reesei; Hc, Histoplasma capsulatum; Bd, Blastomyces dermatitidis*.

A comparative genomics study of 17 Eurotiomycetes revealed an increase in the number of genes encoding aspartyl proteases in *T. marneffei* (unpublished data). Reciprocal BLAST comparisons suggested that *T. marneffei pepA* (PMAA_090410) was least diverged from the *A. fumigatus pep1* gene (Supplementary Table 1). To understand the nature of the expansion, genes found in this family and their orthologues in other species were used to generate a relatedness tree (Figure 1). The 23 genes in the expanded family in *T. marneffei* showed a monophyletic distribution and are therefore all paralogues in *T. marneffei*. The genes were named **p**aralogues **o**f ***p****ep* (*pop*). Long branch lengths in the expansion relative to branch lengths of the *Talaromycete* orthologues point to a rapid generation of variation within the group.

**Figure 2.**
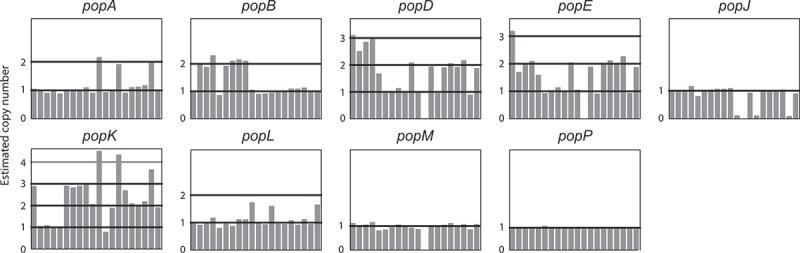
Aspartyl protease-encoding genes vary in copy number between strains. The copy number of *pepA* and the *pop* genes in 20 strains of *T. marneffe*i relative to the reference genome of FRR2161 is depicted. For each gene, the estimated copy number (vertical axis) in each strain (horizontal axis) is shown. Estimated copy number is based on read depth over each gene in each strain. The *popP* gene is an example of a gene with no copy number variation.

In order to assess if changes in gene number were occurring over shorter evolutionary timescales, we examined the intra-species variation within the *pop* expansion. Using the genome sequencing data from a different study, which investigated 20 clinical and environmental isolates of *T. marneffei* (unpublished data), we mapped the depth of reads from these strains for each *pop* gene. This metric can be used as a proxy for the copy number of these genes in each strain (Figure 2). Eight of the 23 *pop* genes showed copy number variation. For example, *popJ* is lost in four strains while *popD, popE, and popM* are lost in one strain each. The *popA, popB* and *popL* genes are duplicated in three, seven and three strains, respectively, while *popD, popE* and *popK* show wide variations in copy number between strains.

### The *pop* genes are functionally conserved despite showing signatures of positive selection

**Figure 3.**
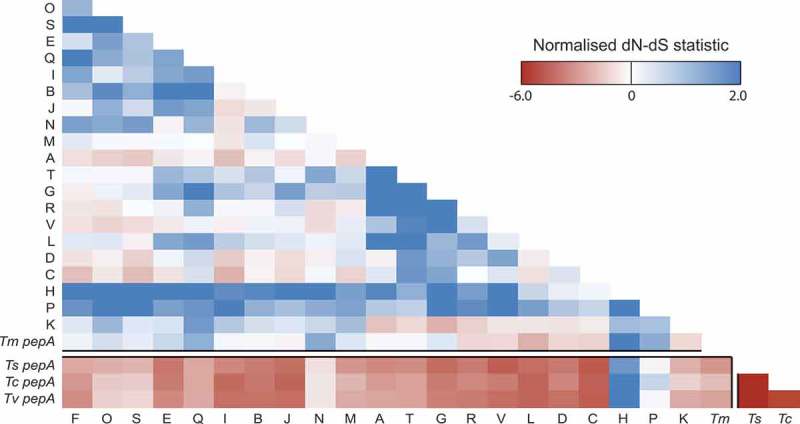
Both positive and negative selection is evident amongst the aspartyl protease-encoding genes. A heatmap showing the normalized non-synonymous/synonymous selection test statistic in pairwise comparison between all *pop* genes and *pepA Talaromycete* orthologues. The axes show the gene identifier (as a one letter abbreviation) for each *T. marneffei pop* gene or the species for each *pepA* gene (*Tm, T. marneffei; Ts, T. stipitatus; Tc, T. cellulolyticus; Tv, T. verruculosus*). Negative values suggest purifying selection is operating while positive values indicate relaxed selection.

To examine the evolutionary forces that have been operating on this gene expansion, we determined the non-synonymous/synonymous (d_N_/d_S_) substitution rates across this family. Codon-based alignments for each of these genes were generated, as were those of their orthologues in three closely related *Talaromyces* species (Figure 3). Selection between *pepA* orthologues in the three *Talaromycetes* is strongly negative indicating a conservation of function in these genes. While negative selection between these three *Talaromyces spp*. genes and the *T. marneffei pop* expansion is still maintained, it is less significant and in the case of *popP* and *popH* is absent. Selection within the *pop* expansion is either neutral or positive. This suggests that these genes are less likely to have a similar biochemical function as their ancestor but conceivably may have evolved new functions.

In order to determine whether this positive selection was confined to particular amino acid residues or domains, the location of variable residues within the expansion was mapped on to the known 3D structure of the *Aspergillus oryzae* orthologue Pep1 [20] (Supplementary Figure 1(a)). This analysis revealed that surface residues were more likely to show variation between individual proteins of the *pop* expansion, while internal residues were significantly more conserved. To quantify the differences in variation between internal and external residues each residue was assigned a score for variation and surface exposure (solvent accessibility). A negative correlation was observed between surface exposure and variation indicating that positive selection occurring in these genes is predominantly associated with the protein surface (Supplementary Figure 1(b)).

Despite the changes in protein sequence due to positive selection the residues and domains that define aspartyl proteases are conserved in the *pop* expansion. These include the pre-pro peptide required for secretion, the activation peptide and the aspartic acid containing active site motifs. The secretion signal and activation peptide are widely conserved in this expansion with some important exceptions (Supplementary Figure 2(a)). The *pepA* product shows a moderate reduction in conservation in both sequences while *popP* and *popH* products show additional residues in the secretion signal as well as differences in the activation peptide compared to the other *pop* proteins. The *popE* activation peptide is disrupted which may impact function. Expression of *popK* has not been detected under any condition tested to date so the lack of conservation in this gene is likely to be unimportant. The core catalytic residues of aspartyl proteases are made up of two DTG or DSG motifs, which are highly conserved across taxa [21] (Supplementary Figure 2(b)). The first of these sites is conserved in 15 of 23 *pop* genes while the second is conserved in 19, with 15 showing conservation of both motifs. Five other proteins show conservative substitutions from G to A in the third residue of the first catalytic core, which is unlikely to affect function. As with the activation and secretion signals of *popK* the catalytic sites of this gene are disrupted. Cysteine residues, which form the structurally important C-terminal disulphide bridge, are absolutely conserved in *pop* proteins.

### The *pop* genes are associated with flanking repetitive sequences

**Figure 4.**
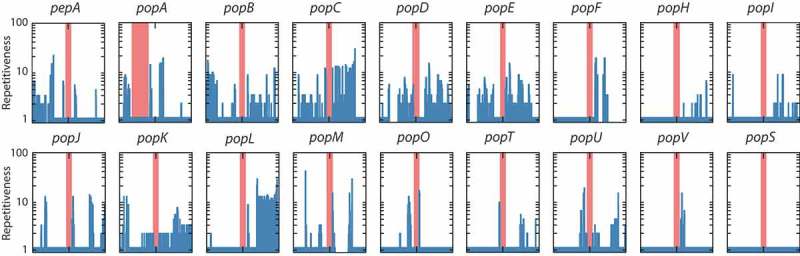
High prevalence of repetitive sequences flanking the aspartyl protease-encoding genes. The prevalence of repetitive sequences in the upstream and downstream 10kb regions flanking *pepA* and *pop* genes is depicted. The red bar denotes each gene locus while the repetitiveness of flanking regions is shown by blue bars. A log scale of 1 to 100 indicates repetitiveness scores for the flanking regions. For example, a score of 5 at a given position indicated that the 30mer at that position is found in five other locations in the genome. The Y-axis is logarithmic. The *popA* gene is offset from the center due to its proximity to the end of a genome assembly scaffold. Repetitiveness of zero is due to unknown sequences in the incomplete scaffold assembly. The *popS* gene is an example of a gene with no repetitive flanking sequences.

In order to understand the mechanism for the expansion of these genes in *T. marneffei* and the variation in copy number between strains, repetitive sequences were examined in the flanking regions surrounding each *pop* gene. In 17 of 23 *pop* genes, sequences within 10 kb of the genes were found elsewhere in the genome (Figure 4). In many cases, more than 10 other copies of the duplicated sequence were identified. Longer regions of duplicated DNA are often associated with transposable elements. Further analysis of these regions identified a number of different transposable element (TE) families including copia and gypsy retrotransposons as well as hAT and mariner DNA transposons, while other repetitive regions were not associated with any known TE (Table 1). Therefore, no single transposon family was uniquely associated with the expanded *pop* family. However, one clade of 10 *pop* genes lack introns entirely and are likely to have formed through an RNA-mediated retrotransposition event (Figure 1, event indicated by black arrowhead). As well as being associated with transposons *pop* genes are also over-represented in subtelomeric regions, with 39% of *pop*s (9 of 23) located less than 100 kb from genome scaffold ends, while this was true for only 5.3% of all genes (594 of 10,036).

**Table 1.** Transposons and repetitive elements in 10kb region surrounding the *pop* genes.

Homologue	5ʹ flanking region	Transposon type	3ʹ flanking region	Transposon type
*pepA*	1.2kb repetitive DNA	-	Gypsy 3–1 AF (+1000bp)	Retrotransposon
*popH*	Mariner 1-AF	DNA transposon	-	-
*popO*	1kb repetitive DNA	-	200bp repetitive DNA	-
*popP*	-	-	-	-
*popS*	Mariner 1-AF	DNA transposon	-	-
*popA*	flanking simple repeats	-	flanking simple repeats	-
*popB*	Copia-1 AT e-1	Retrotransposon	I-5 AO	Retrotransposon
*popC*	Copia-1 Va-1	Retrotransposon	Mariner 1-AF	DNA transposon
*popD*	*popE* gene directly upstream	-	Ribosomal RNA encoding gene	
*popE*	*popD* gene directly upstream	-	Copia 71 BG1	Retrotransposon
*popF*	Copia 71 BG1	Retrotransposon	-	-
*popG*	Mariner fragment	DNA transposon	-	-
*popI*	-	-	2.5kb repetitive DNA	-
*popJ*	flanking simple repeats	-	I-5 AO	Retrotransposon
*popK*	-	-	hAT-1 AN	DNA transposon
*popL*	-	-	1 kb repetitive DNA Copia-1 VA1	Retrotransposon
*popM*	I-5 AO	Retrotransposon	100bp repetitive DNA	
*popN*	hAT 1-AN	DNA transposon	-	-
*popQ*	-	-	-	-
*popR*	2× Gypsy (3–1 AF and 5PGr-1)	Retrotransposon	-	-
*popT*	-	-	200bp repetitive DNA	-
*popU*	I-5 AO	Retrotransposon	Mariner 1-AF	DNA transposon
*popV*	flanking simple repeats	-	-	-

“Simple repeats” are local and sequential and identified by repeatmasker.[22]

“Repetitive DNA” are regions identified in more than one location in the genome that is un-annotated.

### *Pop* genes are important for yeast cell morphogenesis during macrophage infection

**Figure 5.**
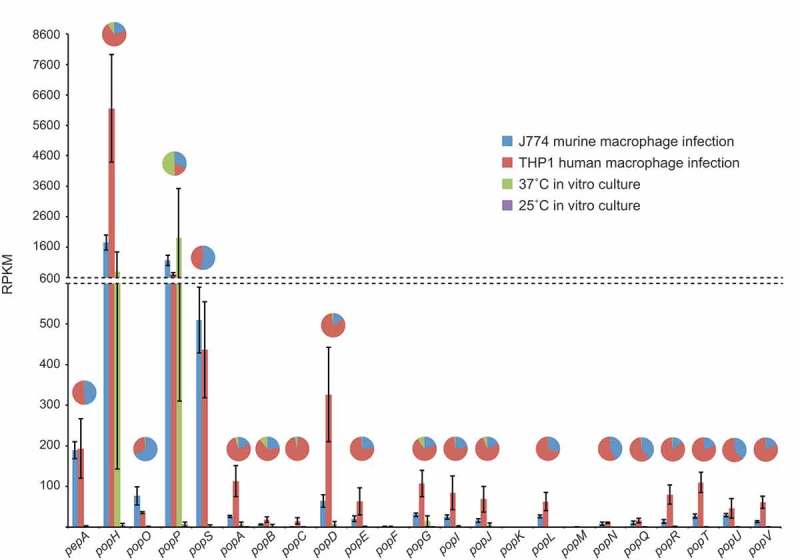
Aspartyl protease genes show specific, differential expression patterns. Gene expression profiles for the *pepA* and *pop* genes during *in vitro* and *ex vivo* growth are shown based on RPKM. The *in vitro* conditions were vegetative growth in the hyphal form at 25 °C and yeast form at 37 °C in BHI medium after four and six days, respectively. The *ex vivo* conditions were growth in the yeast form inside murine J774 and human THP-1 macrophages for 24-h. The pie charts depict a proportional representation of the expression under each condition for each gene. Dashed line denotes a change in the y-axis scale.

In order to determine which, if any, of the *pop* genes are important for pathogenicity of *T. marneffei*, we interrogated a previously generated RNAseq dataset from saprophytic (25°C hyphal *in vitro*) and pathogenic (37°C *in vitro* and *ex vivo* human and murine macrophage infections) growth forms (unpublished data). Expression patterns for the 16 *pop* genes showed a strong bias towards the pathogenic growth form with the remainder showing minimal expression in the conditions tested (Figure 5). In particular, *popP* and *popH* showed strong expression in all conditions with yeast cell morphology. The remaining 14 *pop* genes showed varying degrees of expression specifically during intracellular growth in both human and mouse macrophage lines. In the majority of cases, expression was higher during infection of human cells (Figure 5).

Two groups of genes were selected for molecular genetic analysis based on their expression profiles (Figure 1, genes highlighted in bold). The first group was *popP* and *popH* which show yeast morphology specific expression. The second includes *popO, popS*, and *pepA* which all show *ex vivo* macrophage-specific expression. Deletion strains for each of these five genes were generated using a gene replacement strategy, in order to interrogate the contribution of each to growth and pathogenicity. These strains were also complemented through targeted integration of the wild-type alleles at a specific ectopic genomic locus to verify the mutant phenotype. Double deletion strains were also generated through subsequent rounds of gene replacement. These strains included ∆*popH/*∆*popP*, ∆*popP/*∆*pepA*, ∆*popS/*∆*pepA*, ∆*popS/*∆*popP*, ∆*popS/*∆*popO*, ∆*popO/*∆*pepA*, and ∆*popO/*∆*popP.*

**Figure 6.**
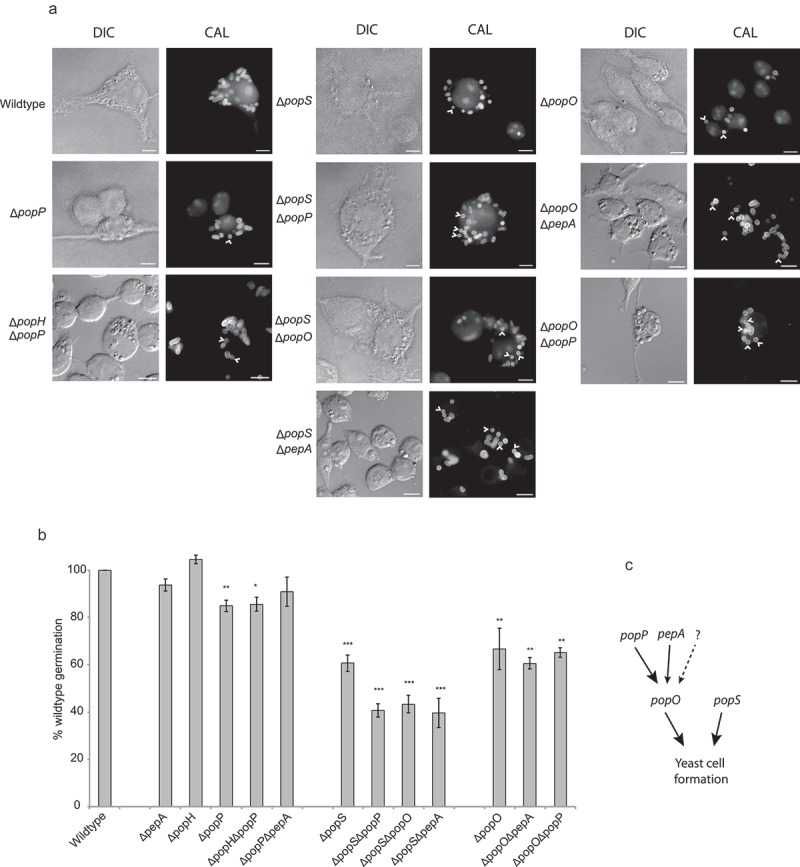
Aspartyl protease-encoding genes are required for appropriate morphogenesis inside host macrophages. (a) Strains bearing mutations in the *pop* genes showing yeast cell-specific expression were used to infect murine J774 macrophages and assayed for yeast cell morphogenesis after 24-h post infection. The *popP, popO* and *popS* single deletion mutant strains show decreased yeast cell formation in macrophages compared to the wild-type. Double deletion mutant strain combinations for these genes show varying levels of reduction in yeast cell formation with *popS* combinations showing the greatest change. Representative images using DIC and epifluorescence (CAL, calcofluor staining) are shown. Arrows highlight spores that failed to produce yeast cells. Scale bars = 10 µm. (b) Conversion of dormant conidia into yeast cells (germination) inside J774 macrophages was quantified for each of the mutant strains. Approximately 100 infected J774 macrophage cells infected with each strain in three biological repeat experiments were used to determine germination, and then plotted as a percentage of the wild-type levels. The error bars represent SEM with t-test values falling in the following range * = <0.05, ** = <0.01, *** = <0.001. (c) A model of the postulated genetic interaction pathway based on the severity of their phenotypes and non-additive, epistatic interactions noted for the various combinations of gene deletions and the single mutants. The dotted arrow and question mark denote possible unidentified pathways or proteases of the *pop* family that may contribute to the formation of yeast cells in *T. marneffei* during intracellular growth.

All strains were examined for phenotypic variation *in vitro* and *ex vivo* to assess the effects during germination and hyphal and yeast growth. No difference in growth, morphology or development was observed for any mutant strain relative to the parental strain *in vitro* (Supplementary Figure 3). At 24-h post infection, wild-type *T. marneffei* within macrophages produced numerous yeast cells that divide by fission. Similar to the wild-type, the ∆*popH* strain showed no phenotypic effect while the ∆*pepA* strain had a minor but insignificant effect during growth in J774 murine macrophages (Figure 6). In contrast, the ∆*popP*, ∆*popS* and ∆*popO* mutants showed a reduction in yeast cell formation to 85.0 ± 2.4%, 60.7 ± 3.5%, and 66.8 ± 8.8% of wild-type levels, respectively. The complemented strains for each of these single mutants restored wild-type levels of yeast cell formation (Table 2). As *popH* and *popP* have similar expression profiles, we examined the possibility of synthetic phenotypes in the double ∆*popH/*∆*popP* mutant. Yeast cell formation in the double mutant was 85.6 ± 3.0% of the wild-type and not significantly different from the ∆*popP* mutant. Similarly, *pepA* mutants lacked significant effects on germination in infected macrophages, despite showing *ex vivo* specific expression, and when examined in combination with ∆*popO* (∆*popO/*∆*pepA*) showed no significant difference from the ∆*popO* single mutant (60.7 ± 2.4% compared to 66.8 ± 8.8%). In contrast, the ∆*popS/*∆*popP* and ∆*popS/*∆*popO* mutants showed additive to near-additive effects (40.7 ± 2.8% and 43.4 ± 3.7%, respectively) while the ∆*popS/*∆*pepA* mutant exhibited synergistic effects (39.6 ± 6.2%) with respect to the single ∆*popS* mutant (60.7 ± 3.5%). The ∆*popO/*∆*popP* double mutant shows no significant difference from ∆*popO* (65.2 ± 2.0% compared to 66.8 ± 8.8%) in yeast cell formation despite the fact that both single mutants show significant independent effects. These data clearly demonstrate a complex relationship with both overlapping and unique functions for these *pop* genes.

**Table 2.** Germination of *pop* mutants and complemented strains.

Strain	Germination (%)^a^	t-Test^b^
wild-type	100 ± 0	^c^
Δ*pepA*	93.79 ± 2.5	
Δ*pepA pepA*^+^	103.17 ± 4.4	
Δ*popH*	104.53 ± 1.9	
Δ*popH popH*^+^	104.05 ± 5.3	
Δ*popP*	84.96 ± 2.4	**
Δ*popP popP*^+^	93.54 ± 2.5	
Δ*popO*	66.78 ± 8.8	**
Δ*popO popO*^+^	123.29 ± 14.2	
Δ*popS*	60.70 ± 3.5	***
Δ*popS popS*^+^	117.67 ± 11.2	
Δ*popH* Δ*popP*	85.59 ± 2.3	*
Δ*popP* Δ*pepA*	90.90 ± 6.2	
Δ*popS* Δ*popP*	40.69 ± 2.8	***
Δ*popS* Δ*popO*	43.37 ± 3.7	***
Δ*popS* Δ*pepA*	39.63 ± 6.2	***
Δ*popO* Δ*pepA*	60.66 ± 2.4	**
Δ*popO* Δ*popP*	65.20 ± 2.0	**
Δ*pepA pepA::mCherry*	110 ± 1.8	
Δ*popH popH::mCherry*	107 ± 2.0	
Δ*popP popP::mCherry*	111 ± 1.5	
Δ*popO popO::mCherry*	103 ± 2.5	
Δ*popS popS::mCherry*	116 ± 4.8	

^a^ Represents the average percent germination relative to the wild-type with the standard error of the mean.

^b^ The t-test values represent the following ranges: * = <0.05, ** = <0.01, *** = <0.001.

^c^ The actual wild-type yeast cell formation percentage was 84.3 ± 0.9%.

### Aspartyl proteases show specific cellular localization

**Figure 7.**
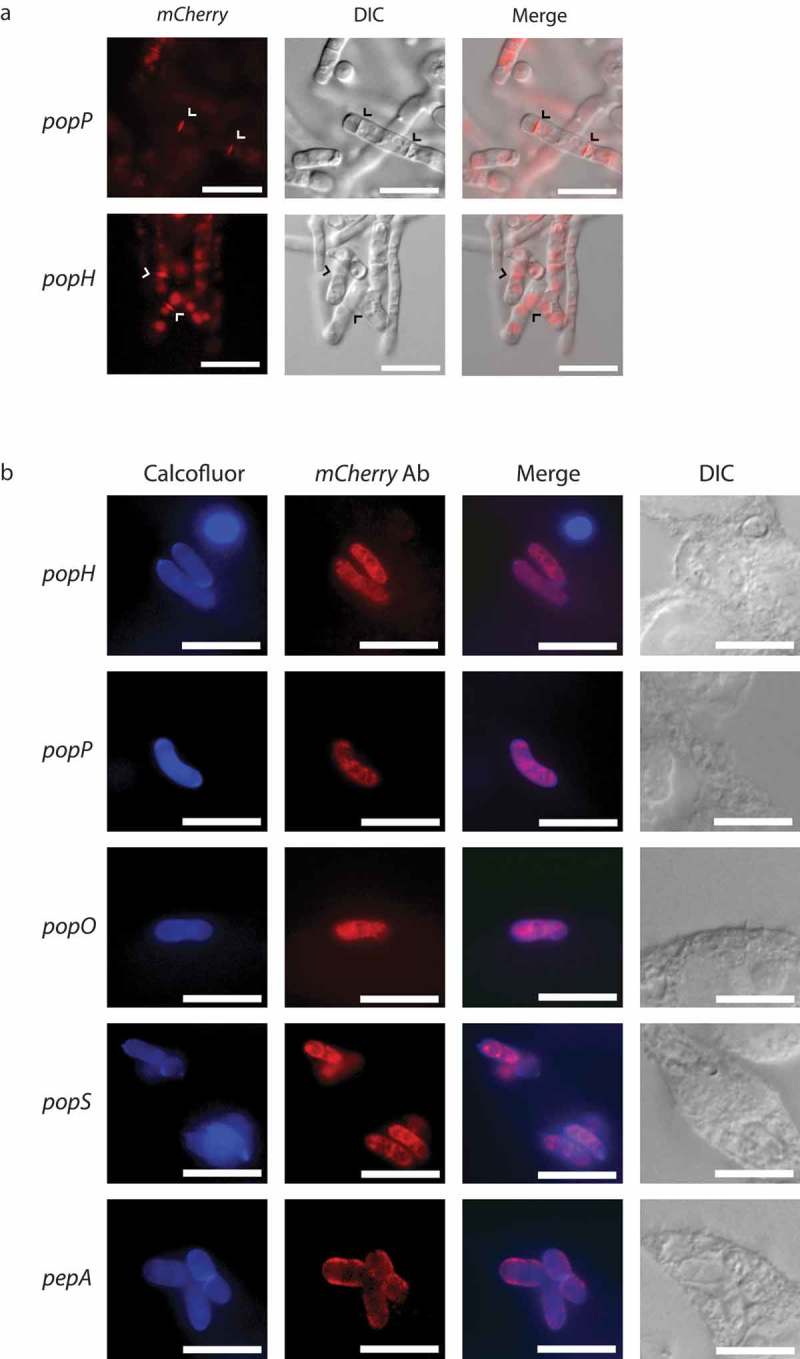
Yeast-specific aspartyl proteases show distinct cellular localization. Full length, C-terminal fusions of the yeast-specific *pepA* and *pop* genes were created with *mCherry* and introduced into the respective deletion strains. (a) During *in vitro* growth of yeast cells at 37°C on BHI medium for 6 days, clear mCherry-based fluorescence was evident for the *popP* and *popH* fusions only and showed both vesicular and septal localisation (arrowheads). Representative images using DIC and epifluorescence are shown. Scale bar = 10 μm. (b) During growth inside J774 macrophages at 24 hours post-infection, immunofluorescence microscopy using antibodies to mCherry shows that the Pop::mCherry fusion proteins are localised as puncta, often, but not exclusively, perinuclear while the PepA::mCherry fusion protein shows clear cell membrane and/or cell wall localisation. Representative images of cells under DIC and after staining with calcofluor (cell walls) and anti-mCherry antibody (fusions) using epifluorescence are shown. Scale bar = 10 μm.

To begin to address the complexity in the functional relationship of the different *pop* gene products, carboxyl-terminal mCherry fusion constructs driven by the native promoter were generated and used to transform the respective deletion strains. Phenotypic characterization of these strains suggested that the fusion alleles complemented the mutant phenotype (Table 2). Using live cells grown *in vitro*, strong fluorescence was evident for strains carrying the *popP* and *popH* fusions during yeast growth at 37°C, while strains with the *popO, popS* and *pepA* fusions showed no fluorescence under these conditions (Figure 7(a)). No fluorescence was detected from any strains grown *in vitro* during hyphal growth at 25°C. These results were entirely consistent with the expression profile data. When these strains were used to infect J774 macrophages, none of the five strains showed detectable fluorescence. To increase the sensitivity of the assay, cells growing inside J774 macrophages were fixed and used for immunofluorescence microscopy with an anti-mCherry antibody. Under these conditions, all five strains showed clear expression of the mCherry fusions.

During *in vitro* yeast growth, the PopH::mCherry and PopP::mCherry proteins showed localization to the septa of undivided arthroconidial cells as well as to internal vesicles (Figure 7(a)). During *ex vivo* growth in J774 macrophages, immunofluorescence in the PopH::mCherry and PopP::mCherry strains appeared as a perinuclear ring as well as in diffuse patches within the cytoplasm, which suggests an association with the secretory pathway (Figure 7(b)). Similar localization was evident for the PopO::mCherry and PopS::mCherry strains. In contrast, the PepA::mCherry strain showed strong co-localization with calcofluor staining, indicating localization to the cell membrane and/or cell wall. Despite the prediction of secretion signals for all proteins, no extracellular fluorescence was detected either *in vitro* or *ex vivo*.

### Aspartyl proteases act cell non-autonomously during macrophage infection

In order to determine if the absence of mCherry fluorescence in the macrophage intracellular environment during infection was due to a lack of secretion or weak signal detection, co-infection experiments with wild-type, ∆*popS*, and ∆*popO* mutants were conducted. As single deletion mutant strains possess intact alleles of the remaining *pop* genes co-infection of macrophages with conidia from two different mutant strains should allow for the production of a full complement of protease gene products in the co-infected macrophage, if the proteases are acting in a cell non-autonomous manner. Macrophages were co-infected with equal numbers of conidia from the ∆*popO*, ∆*popS*, and wild-type strains in all pairwise combinations and yeast cell formation rates were determined at 24-h post infection. Infections with the individual wild-type, ∆*popS* and ∆*popO* strains were also conducted at the same time. Similar to previous observations the ∆*popS* and ∆*popO* mutant strains showed a significant reduction in yeast cell formation (65.9 ± 0.1% and 63.3 ± 2.6%, respectively) compared to wild-type (Table 3). However, the co-infections of ∆*popS* and ∆*popO*, wild-type and ∆*popS* and wild-type and ∆*popO* were comparable to infections by the wild-type alone with no significant reduction in rates of yeast cell formation (96.90 ± 2.5, 102.22 ± 3.5 and 100.67 ± 2.3, respectively) (Table 3). The data clearly indicate that the activity of these genes is cell non-autonomous.

**Table 3.** Germination of *pop* mutants and wild-type in single and co-infection conditions.

	Strain	Germination (%) ^a^	t-Test ^b^
Single infections	wild-type	100 ± 0	^c^
	Δ*popS*	65.89 ± 0.1	***
	Δ*popO*	63.26 ± 2.6	*
Co-infections	Δ*popS*/Δ*popO*	96.90 ± 2.5	
	Wild type/Δ*popS*	102.22 ± 3.5	
	Wild type/Δ*popO*	100.67 ± 2.3	

^a^ Represents the average percent germination relative to the wild-type with the standard error of the mean.

^b^ The t-test values represent the following ranges: * = <0.05, ** = <0.01, *** = <0.001. No asterisks represent conditions that are not significantly different from wild-type.

^c^ The actual wild-type yeast cell formation percentage was 85.71 ± 2.5%.

To examine the secretion of these proteases by *T. marneffei* during macrophage infection, western blot analysis was used to identify the presence of these proteins in fractions of *T. marneffei* infected macrophages (see Materials and Methods). J774 macrophages were infected with conidia from each of the *pepA::mCherry, popP::mCherry, popH::mCherry, popS::mCherry* and *popO::mCherry* strains and protein was extracted from purified yeast cells isolated from within macrophages and the remaining macrophage lysate lacking *T. marneffei* yeast. A strain carrying a histone H1 mCherry fusion (*H1::mCherry*) allele was used as a control. Western blot analysis revealed the presence of the H1::mCherry protein in the *T. marneffei* yeast extract but not in the macrophage fraction (Supplementary Figure 4). In contrast, the Pop::mCherry fusion proteins were detected in both *T. marneffei* yeast and macrophage factions. This was most clearly evident for the *popP::mCherry* strain (Supplementary Figure 4), consistent with the expression profiling data that showed the *popP* gene has the highest expression of all the *pop* family members. A higher level of PopP::mCherry fusion protein is observed in the *T. marneffei* yeast cell faction. Coupled with the co-infection results, these data strongly suggest that at least a number of the proteases encoded by the *pop* gene family are secreted and function outside of *T. marneffei* cells.

## Discussion

The evolution of traits that permit exploitation of new environments can proceed by the repurposing of genes with or without loss of the starting function, or the evolution or acquisition of new genes. While the former is constrained if there is selection acting to maintain the ancestral function of a gene, this is not so for the latter. The evolution or acquisition of new genes can occur by horizontal gene transfer or by gene amplification followed by neofunctionalisation. Gene amplification as a mechanism for evolving new traits provides redundancy for maintaining the original function, relaxes selection on individual genes and increases the level of the gene product, which may in itself have selective advantages.

Here we have described the identification of a novel expansion of aspartyl protease-encoding genes in *T. marneffei* that may have been critical in the evolution of pathogenicity. *T. marneffei* is the only identified animal pathogenic species in the *Talaromyces* genus and the only species in the Eurotiomycetes order that exhibits dimorphic switching; an essential trait for intracellular growth. This study showed that the genes in this expansion form one monophyletic clade, with only single orthologues of the proteases present in other species. In most cases, these genes are associated with subtelomeric regions and surrounded by repetitive sequences, including transposable elements, suggesting mechanisms for the genesis of the expansion. Such mechanisms may have included transposon-driven duplication or unequal crossing-over between repetitive sequences. The role of transposons in the evolution of this gene family is further supported by the presence of a sub-clade of *pop* genes that lack introns, and which are likely to have arisen by retrotransposition. Furthermore, these genes vary in copy number amongst *T. marneffei* isolates, indicating an ongoing dynamic process in the evolution of this family. Given these observations, we hypothesized that this family may have evolved early in the acquisition of pathogenicity in *T. marneffei*.

The activity of genes within expanded gene families is associated with virulence in a number of protozoan and fungal species. These include the VAR genes of *Plasmodium falciparum*, the VSG genes of *Trypanosoma bruscei* and the TLO family in *Candida* species [23–25]. In some of these examples, the gene products have diverged from the ancestral form and the expression of individual genes can vary significantly within and between cellular populations. For cell surface-localized gene products, like the VAR and VSG gene families, this leads to antigenic variation in a population, enabling evasion of the host immune system. In *T. marneffei*, variation in *pop* protein sequence showed clear positive selection and was primarily observed in residues exposed to the host. Given it is predicted that *pop* gene products are secreted, this is consistent with the hypothesis that this may provide a source of antigenic variation for *T. marneffei* to allow escape from detection or help modulate immune interactions during infection.

For the *T. marneffei pop* genes, it is not clear if the expression of each gene shows significant cell to cell variation, as would be expected if they were functioning similarly to the VAR and VSG genes, where switching expression from one member of the gene family to another permits immune evasion and affects infection outcome. Expression profiling suggests that this may not be a significant factor in the action of these genes as each gene showed distinct cell type and growth condition dependent patterns. Fluorescent tagging of members of the Pop family confirmed the expression profiles and revealed novel localization for PopH and PopP. Collectively, these data suggest that at least some *pop* genes in this family are more likely to require the enzymatic function of the gene product to assist the growth of *T. marneffei* under various environmental conditions. This does not preclude some or all of these genes also serving a more structural antigenic variation role.

Aspartyl protease expansions are found in a small number of other pathogenic fungi, where they are proposed to influence pathogenicity and infection. In species of the *Candida* genus, the number of secreted aspartyl protease (SAP) genes differ, with larger numbers hypothesized to confer greater pathogenicity [26]. In *Candida albicans*, SAPs are associated with distinct stages of pathogenesis. Mutations in SAP 1–3 show virulence defects in murine vaginitis models while SAP 4–6 are implicated in peritonitis [27,28]. Deletions of several of the genes in *T. marneffei* that are expressed during *ex vivo* macrophage growth (*pepA, popS*, and *popO*) led to reductions in yeast cell formation under these conditions, and combinatorial deletions of these genes showed more severe or synthetic phenotypes. This suggests that these genes play a role in virulence and the strong conservation of the functional domains in these gene products is consistent with the idea that their enzymatic activity is important. Moreover, the strong positive selection signature that was restricted to the *pop* expansion, the partially redundant function for some of these genes and the ongoing variation in copy number amongst *T. marneffei* strains suggests that the biological roles(s) of these genes may be a recent development in the evolution of pathogenicity of *T. marneffei* and that these genes may act as a source of new effectors to allow *T. marneffei* to respond to new environments. Some genes such as *popP* and *popH* showed minor or no phenotypes when deleted despite strong expression and may therefore be functionally redundant with one or more other *pop* genes.

The *pop* gene products have conserved pre-pro peptide sequences suggesting that they are secreted proteins. Localization of PopH and PopP *in vitro* was to the septa of arthroconidial filaments, which go on to produce yeast cells, and dividing yeast cells, suggestive of a role in the division of yeast cells by fission, a process that is unique to *T. marneffei* amongst the Eurotiomycetes. However, single and double mutants of these two genes showed no defects in this process, indicating that their role is non-essential or redundant. Localization of PopP, PopS, PopH and PopO *ex vivo* was perinuclear and vesicular, typically representing secretory machinery such as the endoplasmic reticulum and Golgi. While none of the gene products were detected extracellularly by fluorescence microscopy, we showed that some of these gene products could be detected in macrophage lysates after infection with *T. marneffei*, even after *T. marneffei* had been removed from the infected cells, and that complementation of the mutant phenotypes could be achieved by coinfection of macrophages with two mutant strains. These data strongly support the prediction that the *pop* gene products are secreted and that the lack of detection extracellularly by fluorescence microscopy indicates a sensitivity issue due to either diffusion or degradation of these proteins. This is consistent with the PepA data, which showed localization immediately adjacent to the cell wall, thus likely outside the cell membrane. In *C. albicans* SAP9 is localized to the cell wall and mediates interactions with phagocytic cells of the immune system [29]. Similarly, *pep1*, the orthologue of *pepA* in *A. fumigatus* is classified as an allergen and as such interacts with the host immune system [30]. The *pepA* gene may also mediate intracellular interactions between *T. marneffei* and host immune cells such as macrophages.

Another possible role for PepA is in an activation pathway for secreted *pop* proteins, which need to be proteolytically cleaved for function (Figure 6(c)). Some support for this hypothesis comes from the phenotypes of double deletion mutants. Combinations with *popS* show that it acts independently of other *pop* genes in yeast cell formation. Conversely, *pepA* or *popP* deletions combined with *popO* show epistatic effects suggesting that they act redundantly in one stage of a pathway that also includes *popO*. If this hypothesis is correct, it is more complex because the *popP;pepA* double deletion did not mimic the *popO* single deletion. Given that only five *pop* genes were characterized in depth in this study it is entirely possible that one or more of the other *pop* genes may also contribute to this pathway, thereby compensating for the loss of activity in *popP* and *pepA*.

Overall the findings clearly show that the *pop* gene family contributes to *T. marneffei* intracellular growth, a key aspect to initiation of infection. Despite the large number of genes comprising this family and their potential for functional overlap, the contributions of specific members to the germination of *T. marneffei* inside macrophages appears to be distinct, underscoring the importance of this expanded group. Recent studies have identified other expanded gene families as significant contributors to *T. marneffei* pathogenicity [31] (unpublished data). Further analysis of all members of the expansion is necessary to understand fully the role these proteases play in the pathogenicity of *T. marneffei*. Interestingly, anti-retroviral therapies used in HIV treatment that target the HIV aspartyl protease can inhibit aspartyl proteases of *C. albicans* and have been shown to influence the virulence of this species *in vivo* [32]. If these compounds also inhibit *pop* proteins in *T. marneffei* they may open new avenues of treatment, especially in HIV infected patients where these medications may already be in use.

## Materials and methods

### Bioinformatic analysis

Protein alignments were performed using ClustalW in the MEGA7 software package [33,34]. A relatedness tree was inferred from the alignment using the neighbor joining method [35] with 500 bootstrap replicates [36], branches with less than 50% support were collapsed. Secretion signals and likelihood of secretion were determined using signalP 4.1 [37] with Eukaryotes organism group and default settings. Repetitive sequences were identified using a custom python script, which counted the occurrence of each 30mer in the genome and then scored the repetitiveness of each position in the genome. Transposable elements were classified using RepeatMasker 4.0.5 with RepBase Update 20,140,131 with the species group set to fungi. Read depth analysis was performed using custom python scripts briefly, normalized depth of read coverage over a given gene was used to estimate the number of copies of that gene in an isolate. ClustalW was used to align coding sequences of *pop* genes in order to carry out positive selection analysis in MEGA7. This analysis consisted of a codon-based z-test for positive selection [38]. The d_N_-d_S_ test statistic was used in the final table to allow examination of both positive and negative selection. Alignments for Supplementary Figure 1 were performed using Clustal Omega [39]. These alignments were then used to calculate and map conservation scores onto the *Aspergillus oryzae pepA* protein structure [20] with AL2CO [40]. Protein structure visualization was performed in MacPyMol v1.8 [41]. Surface exposure (solvent accessibility) was calculated with ASAView [42].

### Molecular methods and plasmid construction

*T. marneffei* genomic DNA was isolated as previously described [43]. Southern and blotting was performed with Amersham Hybond XL membrane using [α-^32^P] dATP labeled probe hybridization using standard methods [44]. Sequences of primers are provided in Supplementary Table 2. Deletion constructs were created using the modified Gateway^TM^ method [45]. The deletion constructs of the following three genes were created using pHW7771 containing the pDONR-*pyrGBlaster* cassette. Wild-type PCR product of *pepA* (PMAA_090410) was generated with primers QQ87 and QQ88 and cloned into pBluescript II SK+ to generate pMP7880. To make the deletion construct pMP7886, PCR primers QQ89 and QQ90 were used to generate an inverse PCR product (which removes from -25 to +1409 of *pepA*) for Gateway^TM^ reaction. Wild-type PCR product of *popH* (PMAA_058850) was generated with primers QQ81 and QQ82 and cloned into pBluescript II SK+ to generate pMP7876. To make the deletion construct pMP7881, PCR primers QQ83 and QQ84 were used to generate an inverse PCR product (which removes from -110 to +1328 of *popH*) for Gateway^TM^ reaction. Wild-type PCR products of *popO* (PMAA_062510) was generated with primers QQ98 and QQ99 and cloned into pBluescript II SK+ to generate pHW7877. To make the deletion construct pHW7882, PCR primers QQ100 and RR1 were used to generate an inverse PCR product (which removes from -59 to +1212 of *popO*) for Gateway^TM^ reaction. The deletion constructs of the following gene were created using pHW7772 containing the pDONR-*bar* cassette. Wild-type PCR products of *popS* (PMAA_089390) was generated with primers QQ75 and QQ76 and cloned into pBluescript II SK+^+^ to generate pHW7879. To make the deletion construct pHW7884, PCR primers QQ83 and QQ84 were used to generate an inverse PCR product (which removes from -121 to +1225 of *popS*) for Gateway^TM^ reaction. The deletion constructs of the following gene were created using pHW7773 containing the pDONR-*riboB* cassette. Wild-type PCR products of *popP* (PMAA_065480) was generated with primers QQ93 and QQ94 and cloned into pBluescript II SK+ to generate pMP7878. To make the deletion construct, pMP7883, PCR primers QQ95 and QQ96 were used to generate an inverse PCR product (which removes from -144 to +1979 of *popP*) for Gateway^TM^ reaction. Complementation constructs were generated by ligating the wild-type PCR products of each gene into *EcoCRI* digested pHB7615 (*niaD* targeting vector) to generate pMP7891 (*pepA*), pMP7887 (*popH*), pHW7888 (*popO*), pHW7890 (*popS*) and pMP7889 (*popP*).

The mCherry tagged constructs were generated by inserting promoter and open reading frame sequences of each gene into *SpeI* digested pHW7911 (*mCherry::niaD* targeting vector). These sequences excluded the stop codon and native terminator sequence of each gene. For the *pepA::mCherry* fusion construct, PCR product generated using QQ87 and XX20 was cut with *XbaI* and ligated into *SpeI* digested pHW7911 to generate pMP8062 For the *popH::mCherry* fusion construct, PCR product generated using QQ81 and XX19 was cut with *SpeI* and ligated into *SpeI* digested pHW7911 to generate pMP8058 For the *popO::mCherry* fusion construct, PCR product generated using QQ98 and XX17 was cut with *XbaI* and ligated into *SpeI* digested pHW7911 to generate pMP8059 For the *popS::mCherry* fusion construct, PCR product generated using QQ75 and XX18 was cut with *XbaI* and ligated into *SpeI* digested pHW7911 to generate pMP8061 For the *popP::mCherry* fusion construct, PCR product generated using QQ93 and XX18 was cut with *SpeI* and ligated into *SpeI* digested pHW7911 to generate pMP8060.

### Fungal strains and media

At 25°C strains were grown on *A. nidulans* minimal medium (ANM) supplemented with 10 mM ammonium sulfate ((NH_4_)_2_SO_4_) as a sole nitrogen source. At 37°C strains were grown on Brain Heart Infusion (BHI) medium (3.7% brain heart infusion).

Transformation was performed previously described using PEG-mediated protoplast fusion [43]. Full details of strains used in this study are listed in Supplementary Table 3. The ∆*pepA* (G1004), ∆*popH* (G994) and ∆*popO* (G997) strains were generated by transforming strain G816 (∆*ligD, pyrG1*) [46] with linearised deletion constructs pHW7886 (*pepA*), pHW7881 (*popH*), pHW7882 (*popO*) and selecting for uracil prototrophy. The ∆*popS* (G1002) strain was generated by transforming strain G809 (∆*ligD::pyrG, pyrG1*) with linearised deletion construct pHW7884 (*popS*) and selecting for glufosinate resistance. The ∆*popP* (G999) strain were generated by transforming strain G829 (∆*ligD, riboB::pyrG*) [42] with linearized deletion constructs pHW7883 (*popP*) and selecting for riboflavin prototrophy. The ∆*pepA*, ∆*popH*, ∆*popO*, ∆*popS* and ∆*popP* mutations were complemented by transforming the respective strains with pMP7891 (*pepA*), pMP7887 (*popH*), pHW7888 (*popO*), pHW7890 (*popS*) and pMP7889 (*popP*) to generate ∆*pepA pepA*^+^ (G1005), ∆*popH popH*^+^ (G995), ∆*popO popO*^+^ (G998), ∆*popS popS*^+^ (G1003) and ∆*popP popP*^+^ (G1000) strains. To generate ∆*popP* ∆*popH* (G1007), ∆*popP* ∆*pepA* (G1011), ∆*popS* ∆*popP* (G1013), ∆*popS* ∆*popO* (G1009), ∆*popS* ∆*pepA* (G1012), ∆*popO* ∆*pepA* (G1010) and ∆*popO* ∆*popP* (G1008) double mutants, ∆*pepA*, ∆*popH*, ∆*popO*, ∆*popS* and ∆*popP* single mutant strains were grown on 1 mg/ml 5-FOA supplemented with 10 mM ((NH_4_)_2_SO_4_) and 5 mM uracil to select for the loss of the *pyrG* marker. These were then transformed with the respective linearised deletion constructs corresponding to the combination required for the strain. The mCherry tagged strains were generated by transforming the ∆*pepA*+, ∆*popH*, ∆*popO*, ∆*popS*+ and ∆*popO* strains with circular *pepA::mCherry* (pMP8062), *popH::mCherry* (pMP8058), *popO::mCherry* (pHW8059), *popP::mCherry* (pHW8060) and *popS::mCherry* (pMP8061) constructs into the respective strains, to generate ∆*pepA pepA::mCherry* (G1006), ∆*popH popH::mCherry* (G996), ∆*popO popO::mCherry* (G1061), ∆*popS popS::mCherry* (G1060) and ∆*popO popO::mCherry* (G1001) strains.

### Macrophage infection assay

J774 and THP-1 macrophage infection assay was performed as per the previously described protocol [47]. Briefly, macrophages were seeded at a concentration of 1 × 10^5^ cells/m into a 6 well microtitre tray containing sterile coverslips and 2 mL of DMEM (J774) or RPMI (THP-1) medium and incubated at 37°C for 24 h. Macrophages were activated with 0.1 μg/mL lipopolysaccharide (LPS)(J774) or differentiated by the addition of 32 μM phorbol 12-myristate 13-acetate (PMA)(THP-1) for 24 h at 37°C. Cells were washed with PBS and 2 mL of complete DMEM or RPMI medium containing 1 × 10^6^ conidia was added. A control lacking conidia was also performed. Macrophages were incubated for 2 h at 37°C to allow conidia to be engulfed, washed once in PBS to remove non-phagocytosed conidia and incubated a further 24 or 48 h at 37°C. Macrophages were fixed in 4% paraformaldehyde and stained with 1 mg/mL fluorescent brightener 28 (calcofluor, CAL) to observe fungal cell walls. Mounted coverslips were examined using differential interference contrast (DIC) and epifluorescence optics for cell wall staining and viewed on a Reichart Jung Polyvar II microscope. Images were captured using a SPOT CCD camera (Diagnostic Instruments Inc) and processed in Adobe Photoshop. The numbers of ungerminated conidia, germlings or yeast cells were recorded in a population of approximately 100 macrophages in three independent experiments.

### Protein extraction and western blot analysis

The macrophage infection assay was performed as previously described (46). Briefly, J774 murine macrophages were seeded at 1 × 10^5^ cells/mL in a flask containing Dulbecco’s modified Eagle medium (DMEM) and grown for 2 days. Macrophages were activated with 0.1 μg/mL lipopolysaccharide (LPS), incubated for 24 h and infected with 3 × 10^8^ conidia of *T. marneffei*. After 2 h the unphagocytosed conidia were washed off, fresh DMEM added and incubated for an additional 24 h. The media was removed and RIPA buffer (10mM Tris, pH7.4, 100mM NaCl, 1mM EDTA, 1mM EGTA, 1% Triton X-100, 10% glycerol, 0.1% SDS and 0.5% deoxycholate) containing cOmplete^TM^ Protease Inhibitor (Roche) and 1mM PMSF, was added to lyse the macrophages. The cell lysate was centrifuged at 2000xg and the pellet predominantly consisting of *T. marneffei* yeast cells was separated from the J774 macrophage protein supernatant. *T. marneffei* cells were homogenized in a FastPrep®-24. Approximately 15 μg of each protein extract, as determined by Bradford assay, was used for SDS-PAGE. Western blot transfer was performed using Immobilon-P polyvinylidene difluoride (PVDF) filters (Merck Millipore Ltd). The mCherry-tagged protein was detected using an anti-mCherry monoclonal rat primary antibody (ThermoFisher Scientific) and an anti-rat IgG horseradish peroxidase (HRP)-linked secondary antibody (Cell Signaling Technology). HRP activity was detected using Clarity^TM^ Western enhanced chemiluminescence (ECL) substrate (Bio-Rad) on a Bio-Rad ChemiDoc^TM^ MP imaging system.

### Microscopy

Immunofluorescence localization of the mCherry-tagged strains was performed with an anti-mCherry rat monoclonal primary (Life Technologies) and a goat anti-rat ALEXA 488 secondary antibody (Molecular Probes) using standard protocols [48]. Immunofluorescence microscopy controls using only primary or secondary antibodies as well as an untagged strain were performed to confirm the specificity of the antibodies.
