# Supplementary material for: A unique aspartyl protease gene expansion in Talaromyces marneffei plays a role in growth inside host phagocytes
Source: Virulence. 2019 Apr 11;10(1):277–91. doi: 10.1080/21505594.2019.1593776 (PMC6527018; doi:10.1080/21505594.2019.1593776)
Supplement: Supplemental Material [file kvir-10-01-1593776-s001.pdf]

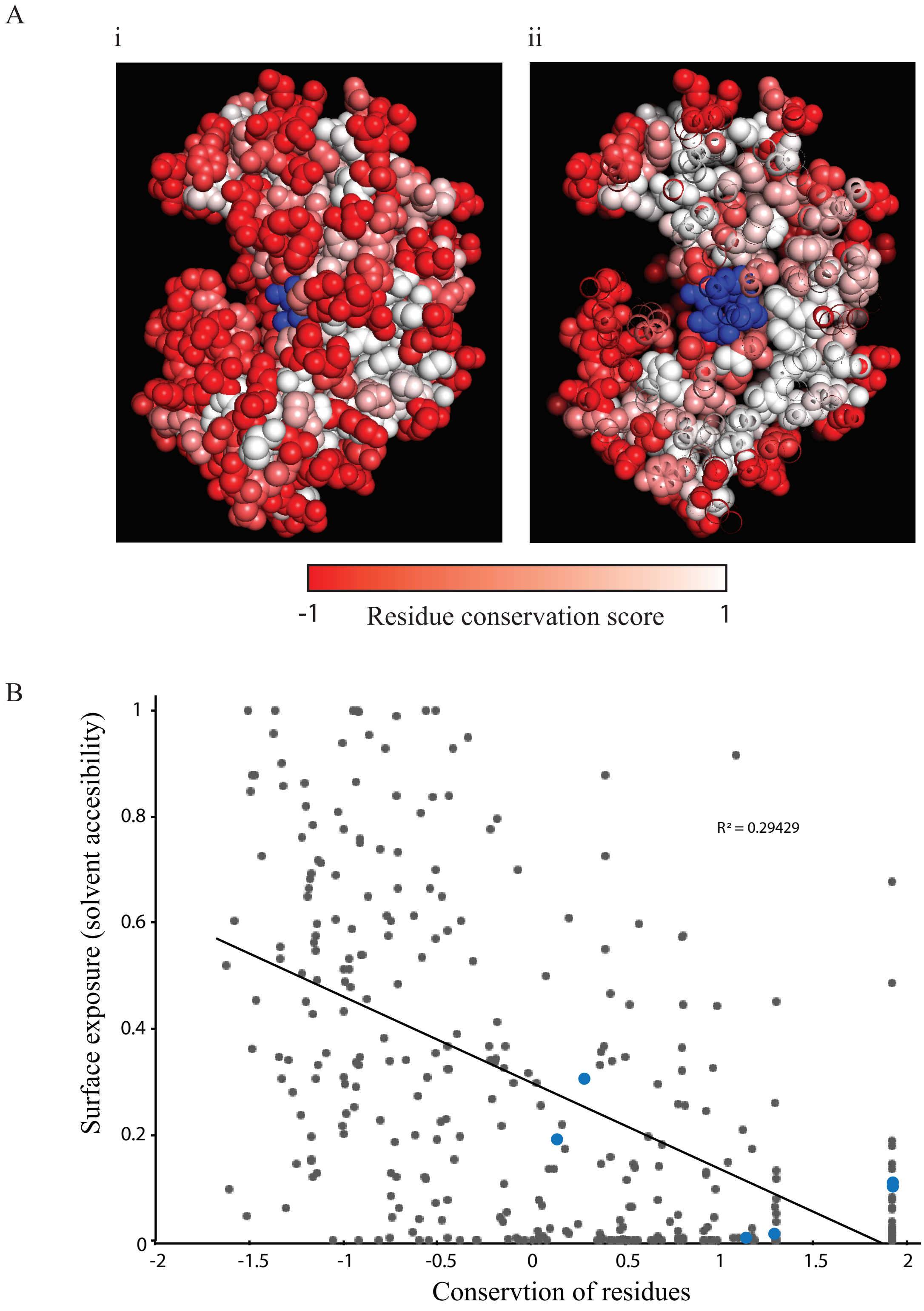

Supplementary Figure 1. Surface residues of the Pop expansion are less conserved.
A. Conservation of amino acid residues within the Pop expansion were mapped onto the structure of the Aspergillus oryzae PepA orthologue using pyMol, allowing visualisation of the relative position of less conserved residues in the protein structure. (i). Surface view of amino acid conservation. (ii). Cross section of structure showing increased conservation of interior residues. Blue regions are conserved 'DTG' catalytic motifs. Gradient from red to white (showing increasing conservation amongst amino acid residues in Pop proteins.
B. Scatterplot of surface exposure (measured by simulated solvent accesibility) compared to conservation of amino acid residues. Higher solvent accessibility score represents greter surface exposure. Residues in blue are the two 'DTG' catalytic motifs with the most conserved pair being the catalytic aspartate residues. This plot shows that a decrease in conservation is correlated with reduced surface exposure.

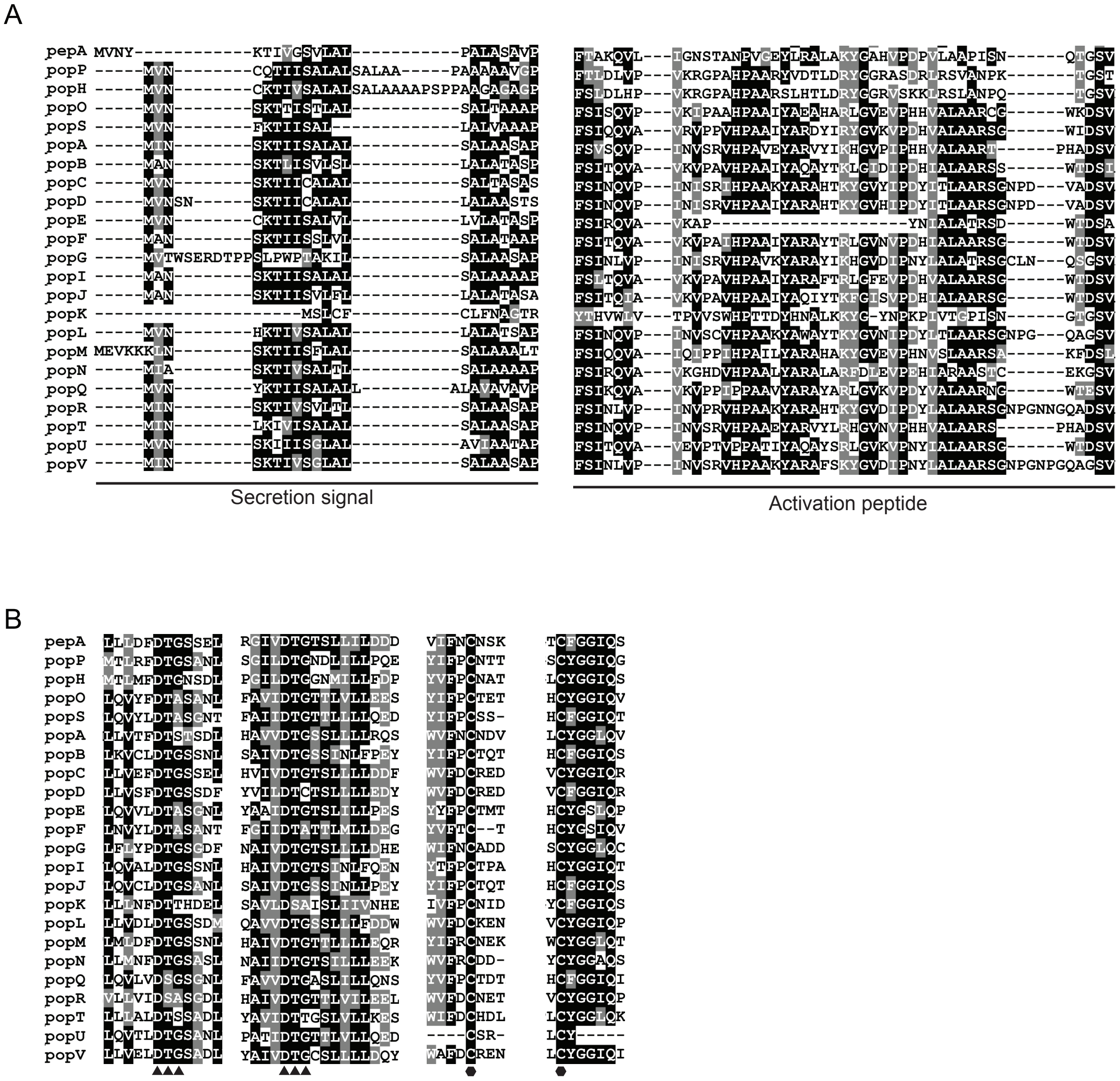

Supplementary Figure 2. Protein sequence alignment of aspartyl protease family in *T. marneffei*.

A. Alignment of the amino terminal end of the predicted *pop* gene products showing the conservation of the pre-pro peptide signal that is necessary for secretion.

B. The N- and C-terminal active sites, represented by the motifs DTG or DSG, are also conserved. The DTG/ DSG motifs (denoted by ▲) contain essential aspartic amino acid residues . Two conserved cysteine residues that are essential for the formation of the C-terminal disulfide bridge are also conserved (denoted by ⬣).

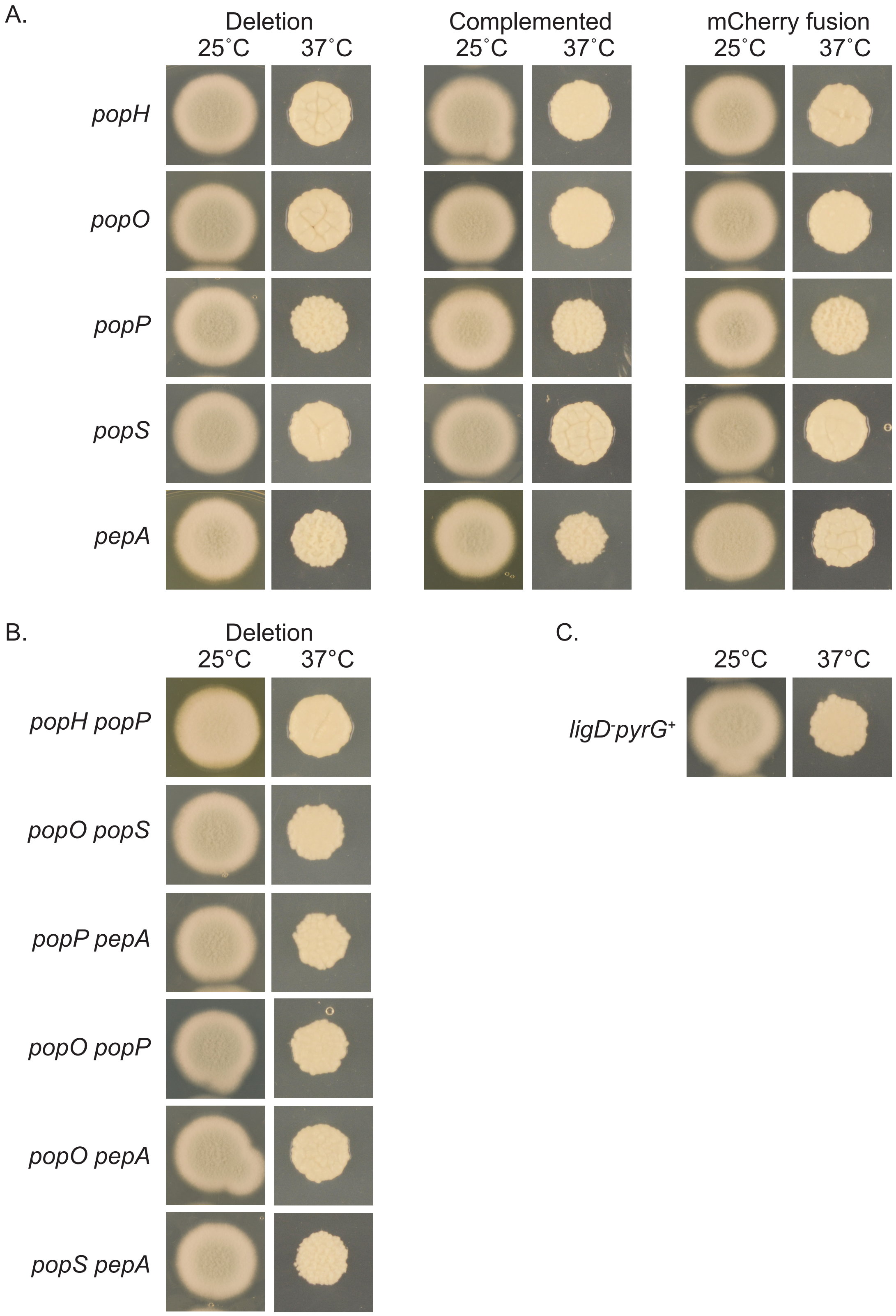

Supplementary Figure 3. Growth of the various genetically modified *pop* strains

Strains were grown for 7 days at 25˚C on ANM medium for hyphal growth and 37˚C on SD medium for yeast growth.

A. Growth of strains with single gene deletions, complemented derivatives of the deletion strains using the wild type allele and complemented derivatives of the deletion strains using the wild type allele fused to the mCherry gene .

B. Growth of strains with double gene deletions of the various genes.

C. Growth of the parental strain used to generate all of the transgenic strains in A and B.

No phenotypic differences was noted between any transgenic strain and the parental strain. *ligD*$^{-}$*pyrG*$^{+}$.

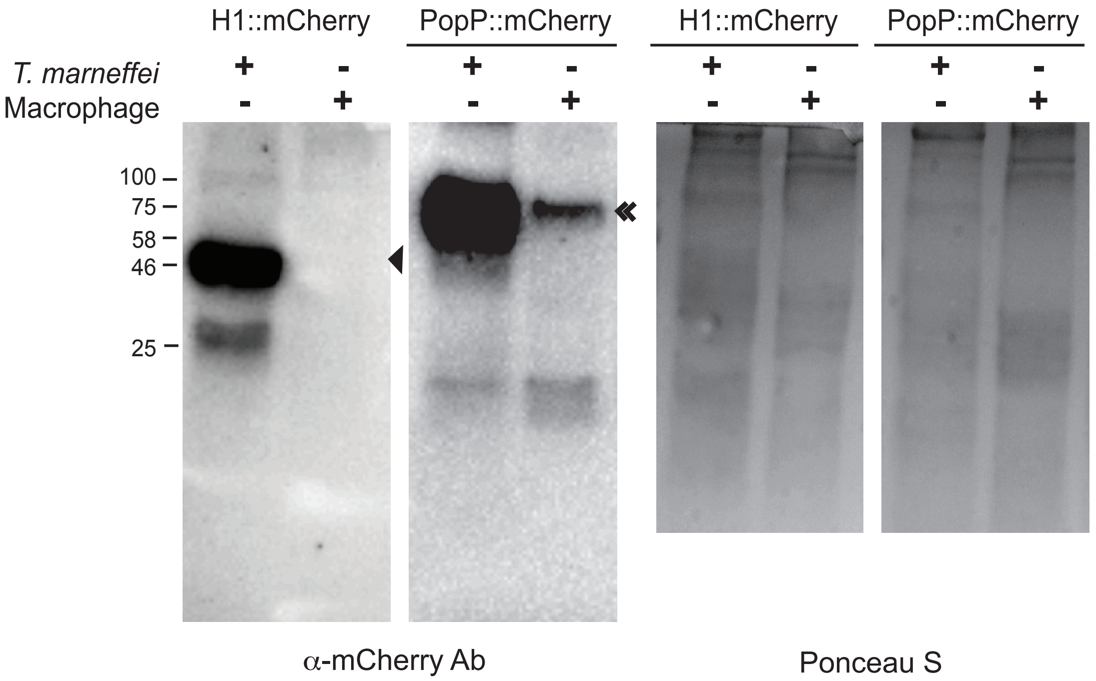

Supplementary Figure 4. Cellular localisation of *T. marneffei* mCherry fusion proteins after infection of macrophages.

J774 macrophages were infected with *T. marneffei* strains carrying either the H1::mCherry or PopP::mCherry fusion construct, grown for 24 hours and cell protein extracts prepared from the internalised *T. marneffei* yeast cells and the the remaining macrophage cell lysates. Western blot analysis using a primary rat anti-mCherry monoclonal antibody (α-mCherry Ab) and secondary anti-rat IgG horseradish peroxidase (HRP)-linked antibody is shown. Molecular mass markers are depicted on the left hand side in kDa. Ponceau S staining was used to check for equivalent loading of proteins for western blot analysis (right hand panels). The *T. marneffei* H1::mCherry fusion protein (~40 kDa) was only detected in *T. marneffei* fraction and not in J774 macrophage fraction (filled arrowhead). The *T. marneffei* Pop::mCherry fusion protein (~72 kDa) was detected in both the *T. marneffei* yeast and J774 macrophage fractions (double arrowhead). The PopP::mCherry protein appears more abundant in the *T. marneffei* yeast cell fraction although this may not truly reflect abundance due to the the highly disparate protein composition of the two extracts.

Supplementary Table 1: Similarity of *A. fumigatus* Pep1 to the aspartyl proteases of *T. marneffei*

Protein [a]	e-value	Percentage identity	Score
PepA	2.00E-118	47.65	885
PopC	9.00E-105	43.92	794
PopR	6.00E-100	43.49	762
PopD	3.00E-99	43.35	757
PopM	3.00E-98	44.44	751
PopL	9.00E-97	44.95	741
PopG	2.00E-94	42.24	726
PopS	5.00E-91	42.02	702
PopF	7.00E-91	40.56	701
PopB	2.00E-90	38.15	699
PopI	5.00E-90	40.11	696
PopK	4.00E-89	43.14	689
PopA	4.00E-89	40.37	690
PopJ	4.00E-89	38.56	690
PopT	1.00E-87	39.73	680
PopV	4.00E-87	40.63	677
PopP	2.00E-85	38.32	665
PopN	2.00E-84	40.7	658
PopH	3.00E-84	39.26	658
PopQ	7.00E-84	40.92	655
PopE	4.00E-79	39.39	621
PopO	2.00E-78	36.7	618
PopU	4.00E-59	37.18	481

[a] – Similarity is based on BLASTp e-value, identity and scores, and the genes are sorted by similarity.

Supplementary Table 2: Primers used in this study

Primer ID	Primer sequence
QQ100	ACCAGGTGTCGATCAGCTTT
QQ75	GCGTACGGGTTCTGGAAAAG
QQ76	CTGACCATGGCGTTTGTGTA
QQ81	TGCATGTAACTGTGTATGGGTGT
QQ82	CAGGTGCATTTTTCTTACGATG
QQ83	GGGGACCCAGCTTTCTTGTACAAAGTGGTTCTAATATCGGCCCATCACC
QQ84	GGGGAGCCTGCTTTTTTGTACAAACTTGTGATTGTGGAACAAAACATTGCT
QQ87	GGAACAATGGCTTGAAGAGG
QQ88	TAGGGCGAGTTTAACGATCC
QQ89	GGGGACCCAGCTTTCTTGTACAAAGTGGTTGGACTAATTGATTGAATGAACG
QQ90	GGGGAGCCTGCTTTTTTGTACAAACTTGTGTCGGTTCTACACGGTCCAA
QQ93	GTGACAGGCTGGCTGTTCCT
QQ94	GCACTGAAACCCCAACACTT
QQ95	GGGGACCCAGCTTTCTTGTACAAAGTGGTTTTTTCAAGCCTGCACTTCC
QQ96	GGGGAGCCTGCTTTTTTGTACAAACTTGTAGGAACTGGGCCGTTAGACT
QQ98	GCCGAAGGTCAACAACAAAG
QQ99	ACAATTGGCGCGATATGATT
RR1	GGGGACCCAGCTTTCTTGTACAAAGTGGTATAGCAGGAACGGAGGGAGT
XX17	TTTTACTAGTGGCCTGGGCCGCGATACCGAT
XX18	TTTTACTAGTGGCCTTGGTGGCGAAACCAAC
XX19	TTTTACTAGTGGCCTTGATGGCGAAGCCAAC
XX20	TTTTTCTAGAAGCCTGAGCAGCCAAACCAAT

Supplementary Table 3: *T. marneffei* strains used in this study.

Strain ID	Full Genotype	Origin
G809	*ΔligD*::*pyrG niaD1 pyrG1*	(Bugeja *et al* , 2012)
G816	*ΔligD*::*pyrG*$^-$ *niaD1 pyrG1*	(Bugeja *et al* , 2012)
G829	*ΔligD*::*pyrG*$^-$ *ΔriboB::pyrG niaD1 pyrG1*	(Bugeja *et al* , 2012)
G994	G816, *ΔpopH::pyrG*	This study
G995	G816, *ΔpopH::pyrG* [*niaD*^{t} *popH*]	This study
G996	G816, *ΔpopH::pyrG* [*niaD*^{t} *popH*::*mCherry*]	This study
G997	G816, *ΔpopO::pyrG*	This study
G998	G816, *ΔpopO::pyrG* [*niaD*^{t} *popO*$^+$]	This study
G999	G829, *ΔpopP::riboB*	This study
G1000	G829, *ΔpopP::riboB* [*niaD*^{t} *popP*]	This study
G1001	G829, *ΔpopP::riboB* [*niaD*^{t} *popP*::*mCherry*]	This study
G1002	G809, *ΔpopS*::*bar*	This study
G1003	G809, *ΔpopS*::*bar* [*niaD*^{t} *popP*]	This study
G1004	G816, *ΔpepA::pyrG*	This study
G1005	G816, *ΔpepA::pyrG* [*niaD*^{t} *pepA*]	This study
G1006	G816, *ΔpepA::pyrG* [*niaD*^{t} *pepA*::*mCherry*]	This study
G1007	*ΔligD ΔriboB niaD1 pyrG1 ΔpopP::riboB ΔpopH::pyrG*	This study
G1008	*ΔligD ΔriboB niaD1 pyrG1 ΔpopP::riboB ΔpopO::pyrG*	This study
G1009	*ΔligD niaD1 pyrG1 ΔpopS*::*bar ΔpopO::pyrG*	This study
G1010	*ΔligD niaD1 pyrG1 ΔpopO::pyrG ΔpepA::pyrG*	This study
G1011	*ΔligD ΔriboB niaD1 pyrG1 ΔpopP::riboB ΔpepA::pyrG*	This study
G1012	*ΔligD niaD1 pyrG1 ΔpopS*::*bar ΔpepA::pyrG*	This study
G1013	*ΔligD niaD1 pyrG1 ΔpopS*::*bar ΔpopP*	This study
G1060	G809, *ΔpopS*::*bar* [*niaD*^{t} *popS*::*mCherry*]	This study
G1061	G816, *ΔpopO::pyrG* [*niaD*^{t} *popO*::*mCherry*]	This study
